# A daily-level, within-person examination of emotion regulation as a mediator of the relationship between sleep and behavior in youth

**DOI:** 10.3389/frsle.2023.1154638

**Published:** 2023-05-16

**Authors:** Paulina T. Feghali, Brooke K. Iwamoto, Olivia M. Triplett, Nicholas J. Rockwood, Timothy D. Nelson, Tori R. Van Dyk

**Affiliations:** ^1^Department of Psychology, Loma Linda University, Loma Linda, CA, United States; ^2^Division of Interdisciplinary Studies, Loma Linda University, Loma Linda, CA, United States; ^3^Department of Psychology, University of Nebraska-Lincoln, Lincoln, NE, United States

**Keywords:** internalizing problems, externalizing problems, sleep duration, sleepiness, emotion regulation

## Abstract

**Objective:**

Youth who experience behavioral and emotional problems are at risk for sleep disturbance, while sleep disturbance also perpetuates behavioral and emotional problems. While the relationship between sleep and psychopathology in clinical mental health samples is well-established, exploration of the underlying mechanisms maintaining this relationship is limited. The purpose of this study is to explore within-person variability in emotion regulation as a mechanism of the relationship between sleep and psychopathology in a clinical youth sample.

**Methods:**

Using a within-person design, 25 children (ages 6–11; 64% male; 44% non-Hispanic White) presenting to outpatient behavioral health treatment with mental health concerns were recruited to participate in a 14-day study. Daily reports of objective sleep duration via actigraphy, self-reported subjective sleepiness, and parent-reported internalizing and externalizing problems and emotion regulation were collected. Multilevel mediation analyses were used to examine the mediating effect of emotion regulation on the daily-level relationship between sleep and behavior problems.

**Results:**

At the within-person level, emotion dysregulation was a significant mediator of the relationships between objective sleep duration and both externalizing [MCCI (0.0005–0.0063)] and internalizing problems [MCCI (0.0001–0.0025)]. Contrary to hypotheses, when youth slept more than usual, internalizing and externalizing problems were worse through the indirect effect of increased emotion dysregulation.

**Conclusions:**

Inconsistencies in schedules and routines, even if in a positive direction, may have short-term negative consequences for youth with emotional and behavioral concerns. Future research should look to address sleep variability and how deviations in routine may impact behavior more broadly, through the indirect effects of emotion regulation.

## 1. Introduction

Prior studies estimate that 20 to 25% of youth in America experience sleep problems, including difficulty in both sleep onset, duration, and quality (Smaldone et al., 2007). It is recommended that school-aged children sleep 9 to 11 h a night (Hirshkowitz et al., 2015). However, over the last 20 years, studies evidence a stable decline in sleep duration by nearly 30–60 min (Taveras et al., 2017), with 17% of 6 to 7-year-olds, 7% of 8 to 9-year-olds, and only 2.5% of 10 to 11-year-old's obtaining the recommended amount of sleep (Gruber et al., 2018). Beyond sleep duration, studies are beginning to emphasize the importance of considering other aspects of sleep health such as self-reported sleep quality when assessing for disordered sleep (Erwin and Bashore, 2017). While young children may not always be consistently accurate reporters of their sleep duration (Bauer and Blunden, 2008), some research indicates that reports of daytime sleepiness are related to short or poor quality of sleep the prior night and predict worse mood and behavior in youth (Fallone et al., 2002). Though poor sleep is common among all youth, there are specific populations that are at even greater risk. For example, youth with preexisting behavioral and emotional problems were found to have a six-fold increase in sleep disturbances compared to their peers (Reigstad et al., 2010). These high rates of sleep problems are concerning considering disturbed sleep has negative effects on cognitive, physical, and emotional functioning (Beebe, 2011).

Although the relationships among sleep (both objective and subjective sleep), mood, and behavior have been well-established in healthy youth (Baum et al., 2014), in clinical mental health samples (Van Dyk et al., 2016), and in sleep-disordered youth samples (Van Dyk et al., 2019a,b), research exploring possible mechanisms maintaining the relationship between sleep and behavior in youth is more limited. Current suggested mechanisms include bedtime behavior problems (Mindell et al., 2006; Alfano et al., 2009; Moore et al., 2011; Tan et al., 2012), biological factors (e.g., genetics, neurotransmitter dysfunction; Serretti et al., 2003; España and Scammell, 2004; Levinson, 2006; Gehrman et al., 2011; Moore et al., 2011), neurobiological processes including amygdala functioning (particularly the impact of REM sleep physiology on amygdala activation; Yoo et al., 2007; Van Der Helm et al., 2011) and prefrontal cortex functioning (i.e., executive funcitoning; Davidson, 2002), hyperarousal (Talley and Shelley-Tremblay, 2020), and emotion regulation (El-Sheikh et al., 2007; Palmer and Alfano, 2017).

**Figure 1:**
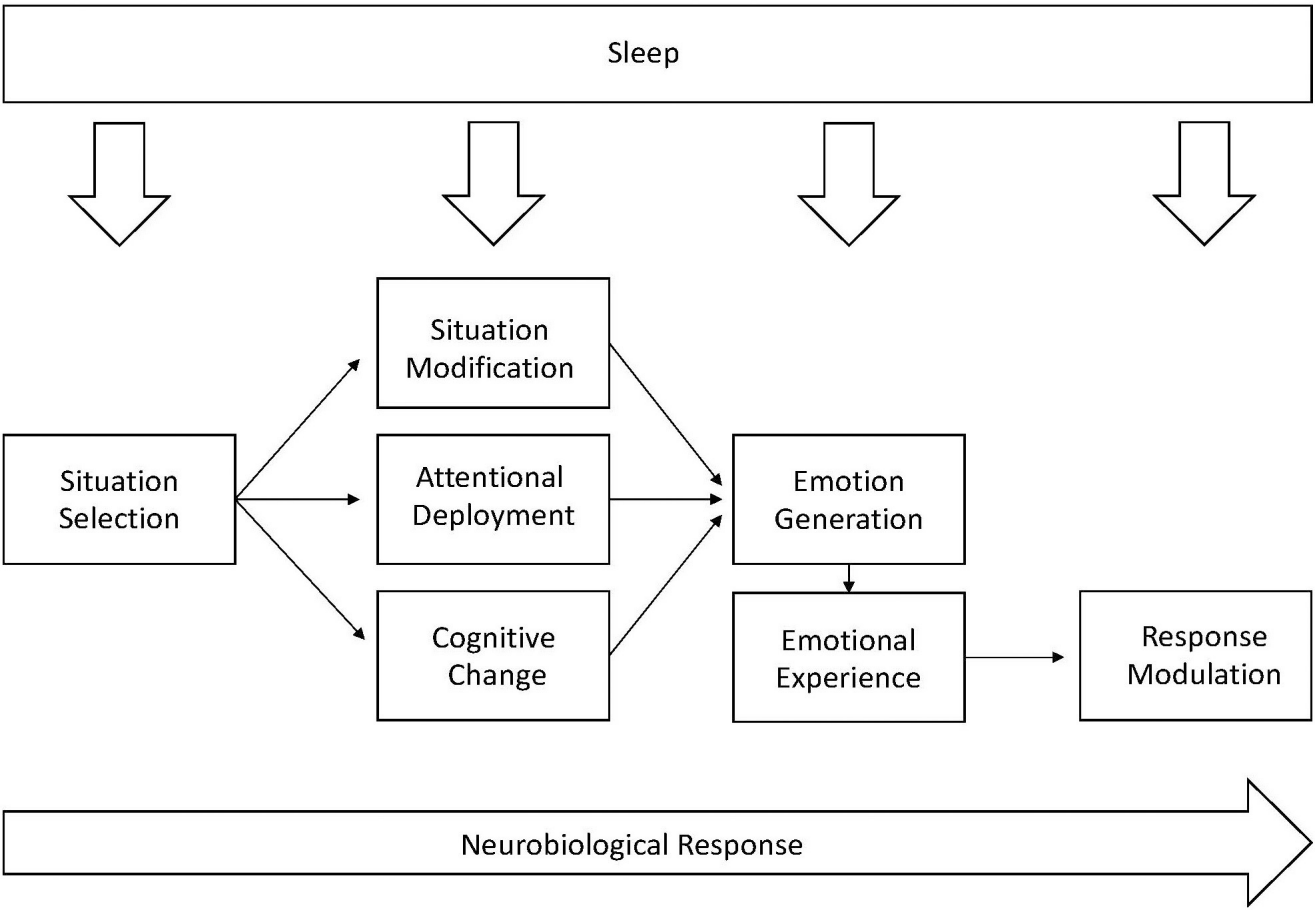
An adaptation of the process model of emotion regulation and the underlying neurological response to emotion. Adapted from Palmer and Alfano (2017).

While it is likely that multiple factors underlie the sleep-psychopathology relationship, emotion regulation remains one of the most frequently posited possible mechanisms (Gross, 1998; Palmer and Alfano, 2017). Conceptually, emotion regulation influences the way individuals generate emotion (Palmer and Alfano, 2017). The current understanding of emotion regulation can be described by using the process model of emotion regulation which includes situation selection, situation modification, attentional deployment, cognitive change, and response modulation. Theoretically, sleep may impact each domain of the process model and, in turn, influences an individual's ability to appropriately regulate emotion (see Figure 1, adapted from Palmer and Alfano, 2017). As outlined in more detail by Palmer and Alfano (2017), sleep-deprived youth may be prone to engage in more emotionally charged situations (situation selection) and subsequently, be more impulsive or have a more difficult time making decisions that would temper an emotional situation (situation modification). Further, when faced with strong emotions, poor sleep may impede the use of appropriate coping strategies such as distraction (attention deployment) and cognitive reappraisal (cognitive change). Finally, once an emotion is generated and experienced, sleep may interfere with the ability to modulate physical and behavioral responses (response modulation). Underlying each aspect of the process model is the neurobiological response (e.g., amygdala functioning) to emotion, which also may be sensitive to sleep (Yoo et al., 2007). Given this theoretical context and prior research suggesting a bidirectional relationship between sleep and behavior in youth, the present study aims to examine changes in emotion regulation as a maintaining factor of the sleep-psychopathology relationship in youth.

In summary, youth presenting to mental health settings may be at increased risk for sleep problems that exacerbate their presenting emotional and behavioral concerns. Further, the current understanding of emotion regulation using the process model indicates that emotion regulation may be a primary underlying mechanism explaining the relationship between sleep and behavior (Palmer and Alfano, 2017). Accordingly, the purpose of this study is to evaluate emotion regulation as a possible mechanism underlying the relationship between sleep and symptoms of psychopathology in a clinical sample of youth with above-average emotional and behavioral problems using longitudinal, daily-level data. By using a daily-level, within-person design, interpretation of effects is focused on how short-term, relative changes in an individual child's functioning (e.g., sleeping less than usual) impact immediate outcomes for that child (e.g., greater emotion regulation or worse behavior than usual) as opposed to between-person analyses which focus more on group effects. Within-person interpretation is particularly relevant for heterogenous clinical samples and provides clinically meaningful information (e.g., how does improving a specific child's sleep impact their short-term functioning in other areas).

Using this framework, it was hypothesized that worse sleep than normal for an individual child (as measured by objective sleep duration and subjective sleepiness) would, on average, be related to worse mood and behavior than normal (as measured by internalizing and externalizing problems). Similarly, it was hypothesized that worse sleep would be related to worse emotion regulation than average and worse emotion regulation would be related to greater internalizing and externalizing problems. Finally, it was hypothesized that emotion regulation would mediate the relationship between daily-level changes in sleep and mood and behavior problems, such that worse sleep than normal would predict relatively worse emotion regulation, which in turn would predict increased mood and behavior problems.

## 2. Methods

### 2.1. Participants

Twenty-five youth ages six to 11 (*M* = 8.67, *SD* = 1.68) and their caregivers were recruited from an outpatient behavior health clinic or a group outpatient behavioral parent-training program. These families were recruited between January 2014 and February 2015. Data from this sample were previously reported by Van Dyk et al. (2016) and is available upon request. Inclusion criteria were: (a) children between the ages of six to 11 years old accompanied by a legal caregiver/parent and (b) the caregiver and child had to have the ability to complete study procedures in English. Exclusion criteria included: (a) child clinical sleep problems (per parent-report), (b) child diagnosed with cognitive/developmental disabilities, and (c) child who did not live with their legal caregiver and/or those who were a ward of the state.

### 2.2. Procedures

The university's Institutional Review Board approved all procedures. Most families were recruited via trained research assistants providing information to outpatient groups (84%) while others were recruited via flyers distributed to parents through the recruitment sources listed above. Interested participants were contacted by telephone to confirm eligibility and schedule the initial in-person session. During this session, both parental consent and child assent were obtained. Youth were asked to wear the actigraph wristwatch all day and night for 14 consecutive days to measure sleep. Both the parents and youth were instructed to complete daily online measures of mental health symptoms, emotion regulation, and sleep. Parents were encouraged to assist young children with reading and responding to questions if needed. Following the 14-day period, families attended a final session to return study materials and collect compensation. Compensation was based on task completion at baseline and throughout the 14-day period. Families were compensated $20.00 for the baseline visit, $5.00 per day (up to 14 days) for completing the daily measures, and $5.00 per day (up to 14 days) for following the actigraph protocol for a total of up to $160.00 at the final session. There was 94% adherence to the daily questionnaire and 96% adherence to the daily actigraph protocol.

### 2.3. Materials

#### 2.3.1. Demographics

Information regarding the age, sex, and ethnicity of their child and additional family socioeconomic information were collected via parent questionnaire.

#### 2.3.2. Youth behavior

The Brief Problems Monitor (BPM; Achenbach et al., 2011) was completed daily by parents. The BPM is a 19-item, evidence-based, brief version of the Child Behavior Checklist (CBCL) used to assess a child's mood and behavior on a daily level. Standardized scores for the internalizing problems and externalizing problems subscales were used in this study. All items used a three-point Likert scale ranging from zero (not true) to two (very true), with higher scores indicative of greater problems. This measure is considered valid and reliable with high correlations with comparative CBCL subscales (Piper et al., 2015).

#### 2.3.3. Emotion regulation

Emotion regulation was assessed via an abbreviated version of The Emotion Questionnaire (Rydell et al., 2003). The abbreviated version is a 4-item, parent-report questionnaire that measures the child's ability to calm down and regulate emotions when they experience emotions such as anger, sadness, and fear. Examples include, “when my child becomes angry, he/she has difficulties calming down on his/her own” and “when my child becomes scared, he/she has difficulties making him/herself calm down.” All items used a five-point Likert scale ranging from one (*Does not apply at all*) to five (*Applies very well to my child*), with higher scores indicative of greater dysregulation. This questionnaire was modified for the current study, such that only four items targeted toward the child's own ability to regulate emotions were included, and items referencing assistance from others to regulate emotion were excluded. While the measure was newly adapted for the present study given the lack of well-established, repeated measures of child emotion regulation, the 4-item questionnaire had strong internal consistency (Chronbach's alpha was 0.809).

#### 2.3.4. Youth sleep

##### 2.3.4.1. Objective sleep duration

ActiGraph wristwatch devices (Actigraph, Pensacola, FL) were used to measure objective sleep for 14 continuous days. Actigraphs are unobtrusive devices worn on the non-dominant wrist that collect child sleep data that have been shown to be comparable to polysomnography (Dayyat et al., 2011). To ensure accuracy of the objective data, parents also completed a daily sleep diary that was referenced when using the actigraphy scoring software. The software converted raw data using well-validated scoring algorithims (Sadeh, 2011) into a daily summary statistic for total sleep duration for nocturnal sleep. For the purpose of this study, total sleep duration (i.e., the total time spent asleep during a sleep episode) was used.

##### 2.3.4.2. Daytime sleepiness

Subjective measurements of sleepiness were collected via the Pediatric Daytime Sleepiness Scale (PDSS). Children responded to 8-items related to daytime sleepiness such as “I needed more sleep” and “I needed help waking up” (Drake et al., 2003). Items were modified in order to capture daily-level responses. For instance, while items in the PDSS are typically rated using a 5-point Likert scale, four items were coded as dichotomous items for daily use (i.e., “how often do you have trouble getting out of bed in the morning” was changed to, “did you have trouble getting out of bed in the morning?”). In addition, two items had an additional response option for, “not applicable” for questions that do not occur daily (i.e., sleepiness during school/when completing homework). The remaining two questions used the original 5-point Likert scale. Given the number of items responded to varied each day, an average score was calculated as opposed to a sum and was used to measure daytime sleepiness with a range of possible scores from 0.33 to 3, with higher scores indicative of more daytime sleepiness. The Cronbach's alpha in the present study was 0.843.

#### 2.3.5. Statistical analysis

First, bivariate analyses of the within-person components of variables (i.e., deviations from person-specific means so as to eliminate dependency in responses provided by the same person) were conducted to determine the relationship between the within-child target variables (i.e., sleep duration, sleepiness, internalizing/externalizing problems, emotion regulation) at the daily-level. To the primary aim, multilevel mediation analyses were conducted using the MLmed macro for SPSS (Rockwood, 2017) to evaluate the indirect effect of emotion regulation as a mediator of the daily-level relationship between both objective sleep and subjective sleepiness and internalizing and externalizing problems. Specifically, analyses examined how an increase/decrease in sleep (relative to a child's own average) was related to a relative increase/decrease in next-day internalizing or externalizing problems through the indirect effect of relative changes in emotion regulation. We controlled for weekday vs. weekend sleep in the mediation analyses. We assessed for outliers and violations of assumptions for multiple mediation, and no outliers or violations were found. Visual inspection of residuals did not demonstrate any major abnormalities.

## 3. Results

### 3.1. Descriptive statistics

Full descriptive statistics on the sample can be found in Table 1. Youth were 64% male and primarily identified as non-Hispanic White. Majority of mothers (80%) and fathers (68%) had at least some college education. Socioeconomically, 48% of families reported an annual income below $50,000 (the median annual income in the area). Detailed descriptive statistics on target variables (i.e., sleep, emotion regulation, externalizing, and internalizing problems) can be found in Table 2. While all parents reported that their children received adequate sleep per night (i.e., >10 h), when objectively measured sleep duration was averaged across the 14-day period, all participants received < 10 h of sleep per night. At the daily level, on average, 36% of participants and 16% of participants were reported to have borderline or clinically significant internalizing problems and externalizing problems, respectively.

**Table 1:** Descriptive statistics on the sample demographics (*n* = 25).

**Demographic variable**	***n***	**%**	***M***	***SD***	**Min**	**Max**
Age			8.67	1.68	6	11
**Sex**						
Female	9	36				
Male	16	64				
**Race/Ethnicity**						
White, non-hispanic	11	44				
African American, non-hispanic	5	20				
White, hispanic	2	8				
American Indian, non-Hispanic	1	4				
Multiracial	6	24				
**Mother's education**						
High school or equivalent	4	16				
Vocational/tech school	1	4				
Some college	9	36				
Bachelor's degree	11	44				
**Father's education**						
Less than high school	1	4				
High school or equivalent	5	20				
Vocational/tech school	1	4				
Some college	6	24				
Bachelor's degree	6	24				
Master's degree	5	20				
Not applicable	1	4				
**Household income**						
Under $10,000	3	12				
$10,000–19,999	1	4				
$20,000–29,999	2	8				
$30,000–39,999	4	16				
$40,000–49,999	2	8				
$50,000–74,999	2	8				
$75,000–99,999	5	20				
$100,000–150,000	5	20				
Over $150,000	1	4				

**Table 2:** Daily descriptive statistics for primary variables.

**Daily variable**	***n*(%)**	***M***	***SD***	**Min**	**Max**	**ICC**
Objective sleep duration (Hours)		7.62	0.60	6.33	8.84	0.230
Under 10 h	25 (100%)					
Pediatric Daytime Sleepiness (PDSS)		1.01	0.50	0.48	2.34	0.633
Emotion regulation (EQ)		6.65	2.95	2.93	13.57	0.462
Internalizing problems		60.82	13.68	50.00	97.29	0.745
Borderline elevated (*T* > 50)	4 (16%)					
Clinically elevated (*T* > 65)	5 (20%)					
Externalizing problems		55.40	6.21	50.14	73.00	0.706
Borderline elevated (*T* > 50)	2 (8%)					
Clinically elevated (*T* > 65)	2 (8%)					

Higher scores on the PDSS indicate greater daytime sleepiness. Higher scores on the EQ indicate worse emotion dysregulation.

### 3.2. Bivariate within-child relationships

See Table 3 for details from bivariate correlations of the within-person components of primary variables. Objective sleep duration was correlated with emotion regulation. Contrary to hypotheses, children who obtained more sleep demonstrated greater emotional dyregulation. As expected, greater emotional dysregulation was related to both greater internalizing and externalizing problems. Finally, internalizing and externalizing symptoms were positively related.

**Table 3:** Bivariate correlations of within-child components of target variables.

**Primary variables**	**Internalizing problems (Parent)**	**Externalizing problems (Parent)**	**Sleep duration (Actigraphy)**	**Daytime sleepiness (Child)**
Emotion dysregulation (Parent)	0.160^**^	0.332^**^	0.134^*^	0.073
Internalizing problems (Parent)	1	0.257^**^	−0.017	0.082
Externalizing problems (Parent)		1	0.104	0.100
Sleep duration (Actigraphy)			1	0.015

^*^p < 0.05, ^**^p < 0.01.

### 3.3. Mediation analyses

To address the primary aim of the study, four multilevel mediation analyses were run to examine the within-child mediating effect of emotion dysregulation on the relationship between (1) sleep duration and externalizing problems, (2) sleep duration and internalizing problems, (3) daytime sleepiness and externalizing problems, and (4) daytime sleepiness and internalizing problems at the daily level. Specifically, we analyzed whether an individual change (more or less) from a child's own average sleep (across 14 days) was associated with higher-than-average internalizing and externalizing behavior problems the next day through the indirect effects of changes in emotion regulation. Given the potential for differences in sleep and behavior according to the day of the week, weekday vs. weeknight was added as a covariate; however, was not significant in any model of the mediation models.

#### 3.3.1. Objective sleep duration and externalizing problems

**Figure 2:**
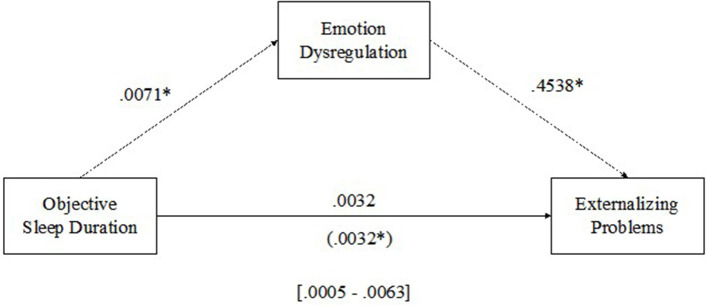
Emotion dysregulation mediating the relationship between sleep duration and externalizing problems. *p < 0.05.

See Figure 2 for results of the multilevel mediation analysis examining emotion dysregulation as a mediator of the relationship between objective sleep duration and externalizing problems. There was a direct positive within-person relationship between objective sleep duration and emotion dysregulation (β = 0.0071, *p* < 0.05) such that as sleep duration increased from a child's own average, emotion dysregulation also increased, and a direct positive within-person relationship between emotion dysregulation and externalizing problems (β = 0.4538, *p* < 0.01) such that as emotion dysregulation increased from a child's own average, externalizing problems increased. The results of level-1 mediation supported an indirect positive relationship between objective sleep duration and externalizing problems mediated by emotion regulation [MCCI (0.0005–0.0063)].

#### 3.3.2. Objective sleep duration and internalizing problems

**Figure 3:**
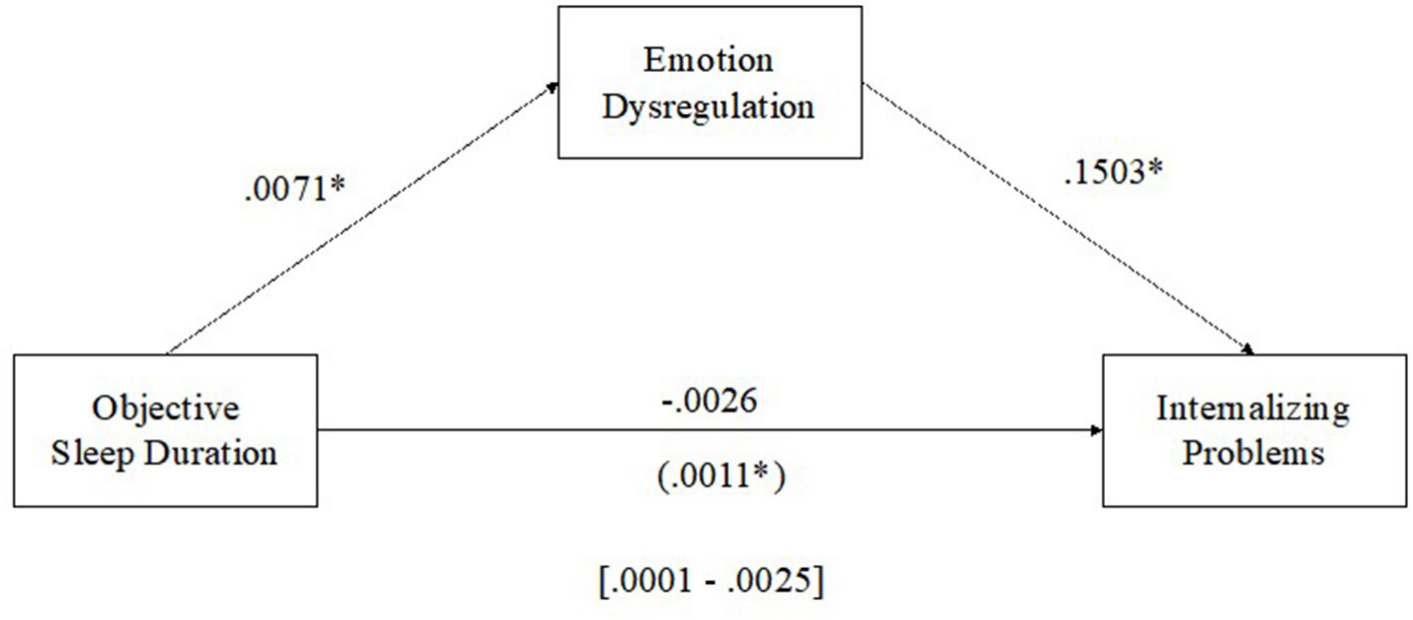
Emotion dysregulation mediating the relationship between sleep duration and internalizing problems. *p < 0.05.

See Figure 3 for results of the multilevel mediation analysis examining emotion dysregulation as a mediator of the relationship between objective sleep duration and internalizing problems. There was a direct positive within-child relationship between objective sleep duration and emotion dysregulation (β = 0.0071, *p* < 0.05) such that as sleep duration increased from average, emotion dysregulation increased, and a direct positive within-child relationship between emotion dysregulation and internalizing problems (β = 0.1503, *p* < 0.01) such that as emotion dysregulation increased from average, internalizing problems increased. The results of the level-1 mediation support an indirect positive relationship between objective sleep duration and internalizing problems mediated by emotion regulation [MCCI (0.0001–0.0025)].

#### 3.3.3. Subjective sleepiness and externalizing problems

**Figure 4:**
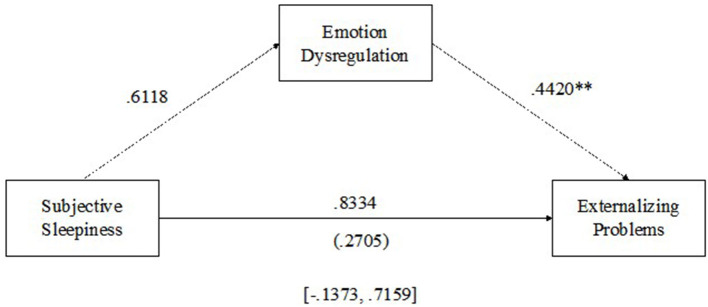
Emotion dysregulation mediating the relationship between subjective daytime sleepiness and externalizing problems. **p < 0.01.

See Figure 4 for results of the multilevel mediation analysis examining emotion dysregulation as a mediator of the relationship between subjective daytime sleepiness and externalizing problems. There were no significant within-person relationships between subjective daily sleepiness and emotion regulation (β = 0.6118, *p* > 0.05) or subjective daytime sleepiness and externalizing problems (β = 0.8334, *p* > 0.05); however, there was a significant positive within-person relationship between emotion regulation and externalizing problems (β = 0.4420, *p* < 0.01) such that as emotion dysregulation increased from average, externalizing problems increased. Emotion regulation was not a significant mediator of the relationship between subjective sleepiness and externalizing problems [MCCI (-0.1373–0.7159)].

#### 3.3.4. Subjective sleepiness and internalizing problems

**Figure 5:**
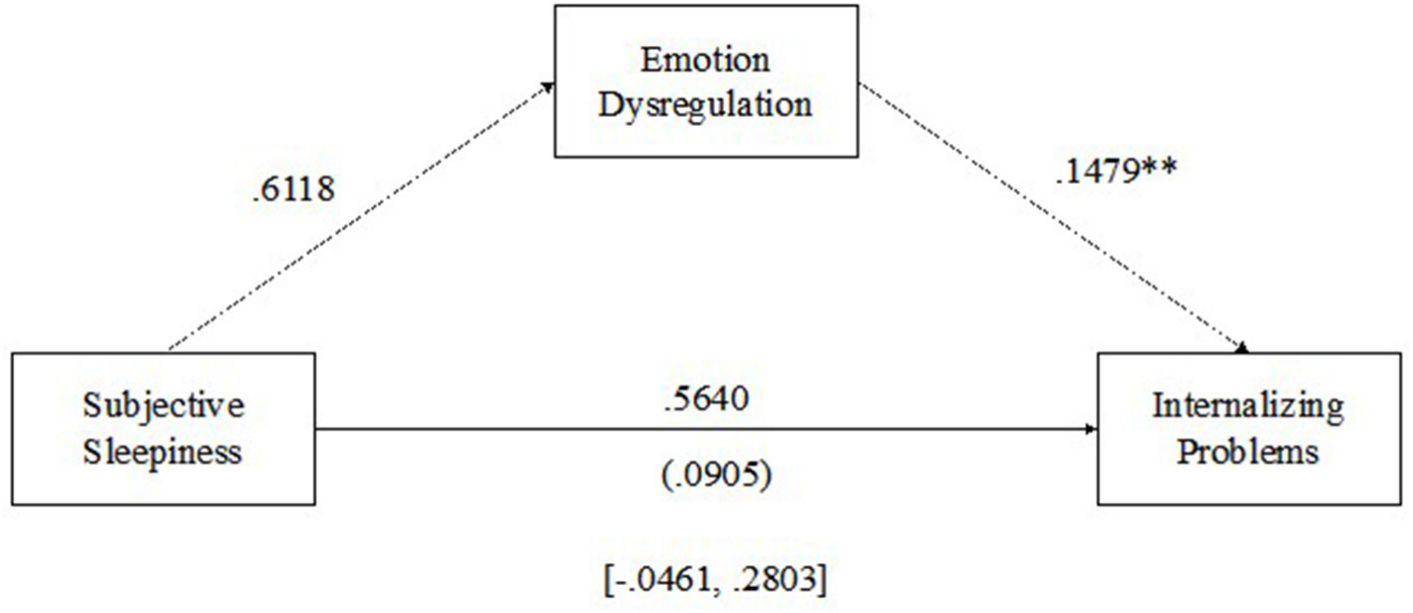
Emotion dysregulation mediating the relationship between subjective daytime sleepiness and internalizing problems. **p < 0.01.

See Figure 5 for the results of the multilevel mediation analysis examining emotion dysregulation as a mediator of the relationship between subjective daytime sleepiness and internalizing problems. There were no significant within-child relationships between subjective daily sleepiness and emotion regulation (β = 0.6118, *p* > 0.05) or subjective daytime sleepiness and internalizing problems (β = 0.5640, *p* > 0.05); however, there was a significant positive within-child relationship between emotion regulation and internalizing problems (β = 0.1479, *p* < 0.05), such that as emotion dysregulation increased from average, internalizing problems increased. Emotion regulation was not a significant mediator of the relationship between subjective sleepiness and internalizing problems [MCCI (-0.0461–0.2803)].

## 4. Discussion

While the results of the present study contribute to the growing body of literature exploring the sleep-behavior relationship in youth, it is important to note that the results may serve as preliminary findings, given the relatively small sample size, small effect sizes, and limitations detailed below. That said, results suggest that emotion regulation may be an underlying mechanism explaining the relationship between sleep duration and both externalizing and internalizing symptoms in a clinical youth sample. However, the directionality of this relationship was opposite of what was hypothesized. Specifically, relative to a child's own “normal” or average, an increase in sleep duration was associated with relatively higher internalizing and externalizing behavior problems through the effects of increased emotion dysregulation. This positive relationship suggests that when children in our sample slept more than usual, they experienced heightened next-day dysregulation, which was associated with higher internalizing and externalizing problems. This relationship may be explained by a change in sleep duration from each individual's baseline (i.e., sleep variability). Given that participants were objectively sleep deprived (i.e., sleeping less than is recommended on nearly every night of the study) a relative increase in sleep may be a more rare occurrence and indicative of a greater disruption in routine which was associated with more dysregulation in the following day, highlighting the importance of maintaining routine for circadian health. Overall, our findings support emerging research which indicates that variability in sleep (sleeping more or less than usual) appears to impact daytime functioning (Becker et al., 2017), although more research examining within-person fluctuations in sleep is needed.

With respect to subjective sleepiness, emotion dysregulation was not a significant mediator of the relationship between self-reported sleepiness and behavior in youth. Interestingly, youth in this sample did not report significant levels of sleepiness on a daily level (range of scores 0.33–3 with higher scores indicating greater sleepiness; average score of 1.01) although their objective measurement of sleep via actigraphy indicated that each participant attained insufficient sleep on most nights. These incongruent findings suggest that child-reported sleepiness, particularly in this younger sample, may not be an accurate proxy of sleep quality perhaps due to poor or limited introspection. Notably, when children do not attain appropriate sleep, excessive daytime sleepiness often manifests as hyperactivity, inattention, and an inability to concentrate on various tasks (Beck and Marcus, 2009). Accordingly, perhaps sleepiness was underreported given the externalizing presentation of subsequent behavior problems. Instead of relying on child-reported sleepiness as an indicator of emotion regulation, mood, or behavior, sleep duration or objective indicators of sleepiness such as a multiple sleep latency test (MSLT) may be a better predictor among young children. Future studies should continue to use objective sleep data, such as actigraphy or MSLT, along with parent-reported perceptions of their child's sleepiness to obtain more accurate data.

In terms of clinical implications, the results have provided preliminary evidence to indicate that although greater emotion dysregulation is significantly related to both greater internalizing and externalizing problems, greater sleep duration than usual was association with an increase in dysregulation at the within-child level. These findings are surprising in that they contradict previously presented theoretical frameworks which suggest that children who sleep less *on average* tend to be more dysregulated, thereby increasing the likelihood of both internalizing and externalizing behaviors. There are several possible explanations as to why this relationship was found. For instance, although youth ages 6 to 11 years old should be sleeping ~9–11 h per night (Hirshkowitz et al., 2015), sleeping a consistent number of hours per night may be just as, or even more important, than sleep duration (Van Dyk et al., 2019c). For example, Van Dyk et al. (2019c) found that when comparing inconsistent sleep to stable sleep using a within-person experimental design, adolescents reported worse outcomes (e.g., increased sleepiness) following a variable sleep schedule vs. a stable sleep schedule, even though average sleep duration was kept constant throughout. Further, a recent meta analysis conducted by Tomaso et al. (2021) found that youth who experience a reduction in total sleep duration across several days tend to evidence a decrease in adaptive emotion regulation, though there were no significant findings in increased maladaptive emotion regulation strategies. This may provide further explanation as to the directionality of the findings of the current study, suggesting that perhaps an increase in mood and behavior problems may be better explained by poor adaptive regulation within the context of decreased or restricted sleep. Though more research is needed in this area with younger youth, if consistency in sleep is just as important as duration, it is possible that a sleep-deprived child will experience worse *short-term* outcomes if they vary from their normal sleep patterns, even if that variation is in a positive direction compared to other children who have consistent sleep patterns. However, an abundance of prior research would suggest that across time, consistently improved sleep would be associated with improved functioning. This highlights the importance of examining within-person differences rather than comparing individual children to their peers, given that changes in one's sleep patterns may have strong implications for overall functioning.

Although the mediated relationship between sleep and behavior yielded unexpected results, the relationship between dysregulation and behavior problems was consistently significant based on within-child analyses. This suggests that providers should focus on emotion regulation when assessing and treating youth with emotional and behavioral problems. Further, prior research suggests that children's behavior is often reliant on their interactions with their parent or caregiver (Davis et al., 2017). For instance, positive parent-child relationships positively impact a child's ability to self-regulate (Davis et al., 2017). As such, it is imperative to recognize the importance of conceptualizing the existing behavioral and emotional concerns of a child within the family system by including parental figures in treatment planning and intervention implementation. Existing evidenced-based interventions, such as behavioral parent training (e.g., Parent-Child Interaction Therapy), are geared toward providing parents with appropriate strategies to assist in managing childhood behavior problems (Bjørseth and Wichstrøm, 2016; Gardner and Leijten, 2017; Thomas et al., 2017; Weber et al., 2019). Using these interventions has been shown to help increase parents' compentency in managing their children's behavior problems, decrease children's behavioral outbursts, and foster an environment that promotes emotional regulation. It is also possible that giving parents tools to manage daytime behavior may generalize to bedtime behaviors that could impact child sleep across the long term.

While, the present study found an unexpected relationship between daily-level, *short-term changes* in sleep duration and behavior, these findings do not overshadow the large body of research that indicates better *overall* sleep is related to long-term improved mood and behavior in youth. Thus, sleep duration should still be improved in sleep-deprived youth using established, evidence-based behavioral sleep strategies in mental health settings, with a particular focus on stabilizing sleep timing and duration. This is pertinent for this particular population, as individuals were recruited through a youth mental health clinic, indicating that these individuals were already seeking behavioral health treatment. Following the assessment of co-morbid sleep disturbances, implementing sleep-related interventions in mental health clinics may be valuable for not only sleep but behavior (Nelson et al., 2016). Furthermore, future intervention research may benefit from building upon the findings of this study, by continuing to examine changes in emotion regulation and behavior across time, particularly within the context of sleep. Collecting longitudinal, consecutive data via the use of both actigraphy and daily behavior diaries would allow for daily-level examination and further exploration regarding the subtle changes in individual mood and behavior as sleep consistency changes.

Although the findings of the current study have contributed to the existing literature proposing emotion dysregulation as a potential mechanism of the sleep-behavior relationship, limitations exist. Of note, the sample size was small. However, due to the use of daily-level data across 14 occasions, there were minimal concerns about adequate power to detect significant findings at the within-child level, which was the primary purpose of this study. However, future studies should look to include a larger, more robust sample to support the current findings and improve generalizability. It is also important to note that these participants were recruited through a mental health facility. To further understand the sleep-behavior relationship within a more general population, future studies should explore these relationships among different populations, particularly among those who are at increased risk of experiencing problems with sleep and/or mental health (e.g., clinical sleep samples). In addition, while the bi-directional nature of the sleep-behavior relationship is well documented, the present study only included sleep as a predictor of behavior. This relationship was examined in this direction in order to apply the Process Model as proposed by Palmer and colleagues in 2017 which focuses on sleep predicting behavior. Future studies should aim to investigate the contrary (i.e., behavior as a predictor of sleep) in order to further understand the role of emotion regulation in the sleep-behavior relationship.

There are several limitations related to measures of target variables used in the present study. For instance, the study used a 4-item adaptation to measure daily emotion dysregulation; however, the measure has not yet been validated as a repeated measures questionnaire. Future studies may consider measuring emotion regulation using physiological (e.g., heart rate, respiration) or performance measures when considering individual reactions to distressing situations. With respect to objective sleep measurement, the present study utilized total sleep duration which provides an important but incomplete picture of objective sleep health. Future studies should also include other objective sleep parameters (e.g., sleep efficiency, wake after sleep onset, MSLT). Further, child-reported sleepiness was not significantly related to objective sleep suggesting that youth in our sample may not have been accurate reporters of their internal states or perhaps were accustom to short sleep and thus did not perceive themselves to be particularly sleepy. Specifically, while children in our sample should have been sleeping 9–11 h per night, objective actigraphy indicated that they were sleeping an average of 7.61 h per night, yet reported low levels of sleepiness. This may imply inaccurate responses to the daily questionnaire or varying perceptions of sleepiness regardless of actual sleep. Alternative approaches to measuring sleepiness (e.g., parent report, objective MSLT) may be more accurate in young children who consistently demonstrate insufficient sleep. More research is needed to better understand sleepiness in children (Fallone et al., 2002).

Finally, considering time precedence when using mediation models is important in order to make strong conclusions related to how the predictor (i.e., sleep) causally influences the mediatior (i.e., emotion dysregulation) and the subsequent outcome (i.e., behavior). While our objective measure of sleep has a clear time precedence with the mediator and outcome, reporting of emotion dysregulation and both internalizing and externalizing problems occurred simultaneously. Perhaps more problematic, the subjective report of sleepiness also occurred simultaneously and may contribute to the failure to detect significant effects within this predictor. While the simultaneous measurement of these variables reduced participant burden related to multiple reporting times within a given day, future research should consider ways to establish time precendence. For example, sleepiness could be reported in the morning with emotion regulation, mood, and behavior reported at the end of the day.

### 4.1. Conclusion

Overall, the purpose of the study was to test a theoretical model presenting emotion regulation as a potential mechanism maintaining the relationship between poor sleep and psychopathology in a clinical youth mental health sample. While our findings did support the theory that the cyclical relationship of sleep and psychopathology is being maintained by emotion regulation, more specifically by dysregulation, the directionality of this relationship was unexpected. Specifically, the findings suggest that a deviation from an individual's average amount of sleep per night, when one is regularly sleep-deprived, may result in increased internalizing and externalizing behaviors through an increase in emotion dysregulation. Of note, these findings are three-fold. First, the results found that an increase in dysregulation was related to increased sleep duration, rather than decreased sleep duration. Second, there was no significant relationship found between perceived daily sleepiness and emotion regulation. Finally, these results were specifically found when examining within-child relationships, suggesting that a child's dysregulation and behavior problems are dependent on their own average objective sleep duration, rather than whether or not they are sleeping more or less than other children their age. These findings have important implications for both research and clinical practice, warranting additional examination of potential mediating mechanisms of the sleep and behavior relationship.

## Data availability statement

The raw data supporting the conclusions of this article will be made available by the authors, without undue reservation.

## Ethics statement

The studies involving human participants were reviewed and approved by University of Nebraska-Lincoln Institutional Review Board. Written informed consent to participate in this study was provided by the participants' legal guardian/next of kin.

## Author contributions

TV and TN contributed to the study conception and design in addition to data collection. PF, NR, and TV organized the database and performed statistical analyses. PF wrote the first draft of the manuscript. BI and OT wrote sections of the manuscript. All authors contributed to the manuscript revision, read, and approved the submitted version.

## References

[B1] Achenbach T. McConaughy S. Ivanova M. Rescorla L. (2011). Manual for the ASEBA brief problem monitor (BPM). Burlington, VT: ASEBA, 1–33.

[B2] Alfano C. A. Zakem A. H. Costa N. M. Taylor L. K. Weems C. F. (2009). Sleep problems and their relation to cognitive factors, anxiety, and depressive symptoms in children and adolescents. Depress. Anxiety 26, 503–512. 10.1002/da.20443 19067319

[B3] Bauer K. M. Blunden S. (2008). How accurate is subjective reporting of childhood sleep patterns? a review of the literature and implications for practice. Curr. Pediatr. Rev. 4. 132–142. 10.2174/157339608784462025

[B4] Baum K. T. Desai A. Field J. Miller L. E. Rausch J. Beebe D. W. (2014). Sleep restriction worsens mood and emotion regulation in adolescents. J. Child Psychol. Psychiatry 55, 180–190. 10.1111/jcpp.12125 24889207 PMC4047523

[B5] Beck S. E. Marcus C. L. (2009). Pediatric polysomnography. Sleep Med. Clin. 4, 393–406. 10.1016/j.jsmc.2009.04.007 20161110 PMC2739664

[B6] Becker S. P. Sidol C. A. Van Dyk T. R. Epstein J. N. Beebe D. N. (2017). Intraindividual variability of sleep/wake patterns in relation to child and adolescent functioning: a systemic review. Sleep Med. Rev. 34, 94–121. 10.1016/j.smrv.2016.07.004 27818086 PMC5253125

[B7] Beebe D. W. (2011). Cognitive, behavioral, and functional consequences of inadequate sleep in children and adolescents. Pediatr. Clin. 58, 649–665. 10.1016/j.pcl.2011.03.002 21600347 PMC3100528

[B8] Bjørseth Å. Wichstrøm L. (2016). Effectiveness of parent-child interaction therapy (PCIT) in the treatment of young children's behavior problems. a randomized controlled study. PLoS ONE 11:e0159845. 10.1371/journal.pone.0159845 27622458 PMC5021353

[B9] Davidson R. J. (2002). Anxiiety and affective style: role of prefrontal cortex and amygdala. Biol. Psychiatry 51, 68–880. 10.1016/S0006-3223(01)01328-2 11801232

[B10] Davis M. Bilms J. Suveg C. (2017). In sync and in control: a meta-analysis of parent–child positive behavioral synchrony and youth self-regulation. Fam. Process 56, 962–980. 10.1111/famp.12259 27774598

[B11] Dayyat E. A. Spruyt K. Molfese D. L. Gozal D. (2011). Sleep estimates in children: parental versus actigraphic assessments. Nat. Sci. Sleep 3, 115–123. 10.2147/NSS.S25676 23616722 PMC3630966

[B12] Drake C. Nickel C. Burduvali E. Roth T. Jefferson C. Badia P. (2003). The pediatric daytime sleepiness scale (PDSS): sleep habits and school outcomes in middle-school children. Sleep 26, 455–458. 10.1037/t02761-000 12841372

[B13] El-Sheikh M. Buckhalt J. A. Mark Cummings E. Keller P. (2007). Sleep disruptions and emotional insecurity are pathways of risk for children. J. Child Psychol. Psychiatry 48, 88–96. 10.1111/j.1469-7610.2006.01604.x 17244274

[B14] Erwin A. M. Bashore L. (2017). Subjective sleep measures in children: self-report. Front. Pediatr. 5:22. 10.3389/fped.2017.00022 28243584 PMC5303893

[B15] España R. A. Scammell T. E. (2004). Sleep neurobiology for the clinician. Sleep 27, 811–820. 10.1093/sleep/27.4.811 15283019

[B16] Fallone G. Owens J. A. Deane J. (2002). Sleepiness in children and adolescents: clinical implications. Sleep Med. Rev. 6, 287–306. 10.1053/smrv.2001.0192 12531133

[B17] Gardner F. Leijten P. (2017). Incredible Years parenting interventions: current effectiveness research and future directions. Curr. Opin. Psychol. 15, 99–104. 10.1016/j.copsyc.2017.02.023 28813277

[B18] Gehrman P. R. Meltzer L. J. Moore M. Pack A. I. Perlis M. L. Eaves L. J.. (2011). Heritability of insomnia symptoms in youth and their relationship to depression and anxiety. Sleep 34, 1641–1646. 10.5665/sleep.1424 22131600 PMC3208840

[B19] Gross J. J. (1998). The emerging field of emotion regulation: an integrative review. Rev. Gen. Psychol. 2, 271–299. 10.1037/1089-2680.2.3.27129128685

[B20] Gruber R. Somerville G. Wells S. Keskinel D. Santisteban J. A. (2018). An actigraphic study of the sleep patterns of younger and older school-age children. Sleep Med. 47, 117–125. 10.1016/j.sleep.2018.03.023 29793184

[B21] Hirshkowitz M. Whiton K. Albert S. M. Alessi C. Bruni O. DonCarlos L.. (2015). National sleep foundation's sleep time duration recommendations: methodology and results summary. Sleep Health 1, 40–43. 10.1016/j.sleh.2014.12.010 29073412

[B22] Levinson D. F. (2006). The genetics of depression: a review. Biol. Psychiatry 60, 84–92. 10.1016/j.biopsych.2005.08.024 16300747

[B23] Mindell J. A. Kuhn B. Lewin D. S. Meltzer L. J. Sadeh A. American Academy of Sleep Medicine. (2006). Behavioral treatment of bedtime problems and night wakings in infants and young children. Sleep 29, 1263–1276. 10.1093/sleep/29.10.1263 17068979

[B24] Moore M. Kirchner H. L. Drotar D. Johnson N. Rosen C. Redline S. (2011). Correlates of adolescent sleep time and variability in sleep time: the role of individual and health related characteristics. Sleep Med. 12, 239–245. 10.1016/j.sleep.2010.07.020 21316300 PMC3050885

[B25] Nelson T. D. Van Dyk T. R. McGinnis J. C. Nguyen A. V. Long S. K. (2016). Brief sleep intervention to enhance behavioral parent training for noncompliance: preliminary findings from a practice-based study. Clin. Pract. Pediatr. Psychol. 4, 176. 10.1037/cpp0000132

[B26] Palmer C. A. Alfano C. A. (2017). Sleep and emotion regulation: an organizing, integrative review. Sleep Med. Rev. 31, 6–16. 10.1016/j.smrv.2015.12.006 26899742

[B27] Piper B. Gray H. Raber J. Birkett M. (2015). Reliability and validity of the brief problem monitor: an abbreviated form of the child behavior checklist. Psychiatry Clin. Neurosci. 68, 759–767. 10.1037/e520582015-011 24735087 PMC4182328

[B28] Reigstad B. Jørgensen K. Sund A. M. Wichstrøm L. (2010). Prevalences and correlates of sleep problems among adolescents in specialty mental health services and in the community: what differs? Nord. J. Psychiatry 64, 172–180. 10.3109/08039480903282392 19883190

[B29] Rockwood N. J. (2017). Advancing the formulation and testing of multilevel mediation and moderated mediation models (Unpublished master's thesis). Columbus, OH: The Ohio State University.

[B30] Rydell A. M. Berlin L. Bohlin G. (2003). Emotionality, emotion regulation, and adaptation among 5-to 8-year-old children. Emotion 3, 30–47. 10.1037/1528-3542.3.1.30 12899315

[B31] Sadeh A. (2011). The role and validity of actigraphy in sleep medicine: an update. Sleep Med. Rev. 15, 259–267. 10.1111/1467-8624.7402008 21237680

[B32] Serretti A. Benedetti F. Mandelli L. Lorenzi C. Pirovano A. Colombo C.. (2003). Genetic dissection of psychopathological symptoms: insomnia in mood disorders and CLOCK gene polymorphism. Am. J. Med. Genet. B Neuropsychiatr. Genet. 121, 35–38. 10.1002/ajmg.b.20053 12898572

[B33] Smaldone A. Honig J. C. Byrne M. W. (2007). Sleepless in America: inadequate sleep and relationships to health and well-being of our nation's children. Pediatrics 119, S29–S37. 10.1542/peds.2006-2089F 17272582

[B34] Talley G. Shelley-Tremblay J. (2020). The relationship between mindfulness and sleep quality is mediated by emotion regulation. Psychiatry Int. 1, 42–66. 10.3390/psychiatryint102000734544298

[B35] Tan E. Healey D. Gray A. R. Galland B. C. (2012). Sleep hygiene intervention for youth aged 10 to 18 years with problematic sleep: a before-after pilot study. BMC Pediatrics 12, 1–9. 10.1186/1471-2431-12-189 23216856 PMC3538572

[B36] Taveras E. M. Rifas-Shiman S. L. Bub K. L. Gillman M. W. Oken E. (2017). Prospective study of insufficient sleep and neurobehavioral functioning among school-age children. Acad. Pediatr. 17, 625–632. 10.1016/j.acap.2017.02.001 28189692 PMC5545152

[B37] Thomas R. Abell B. Webb H. J. Avdagic E. Zimmer-Gembeck M. J. (2017). Parent-child interaction therapy: a meta-analysis. Pediatrics 140:e20170352. 10.1542/peds.2017-0352 28860132

[B38] Tomaso C. C. Johnson A. B. Nelson T. D. (2021). The effect of sleep deprivation and restriction on mood, emotion, and emotion regulation: three meta-analyses in one. Sleep, 44:zsaa289. 10.1093/sleep/zsaa289 33367799 PMC8193556

[B39] Van Der Helm E. Yao J. Dutt S. Rao V. Salentin J. M. Walker M. P. (2011). REM sleep depotentiates amygdala activity to previous emotional experiences. Curr. Biol. 21, 2029–2032. 10.1016/j.cub.2011.10.052 22119526 PMC3237718

[B40] Van Dyk T. R. Becker S. P. Byars K. C. (2019a). Rates of mental health symptoms and associations with self-reported sleep quality and sleep hygiene in adolescents presenting for insomnia treatment. J. Clin. Sleep Med. 15, 1433–1442. 10.5664/jcsm.7970 31596208 PMC6778362

[B41] Van Dyk T. R. Becker S. P. Byars K. C. (2019b). Mental health diagnoses and symptoms in preschool and school age youth presenting to insomnia evaluation: prevalence and associations with sleep disruption. Behav. Sleep Med. 17, 790–803. 10.1080/15402002.2018.1518224 30260686 PMC6526081

[B42] Van Dyk T. R. Thompson R. W. Nelson T. D. (2016). Daily bidirectional relationships between sleep and mental health symptoms in youth with emotional and behavioral problems. J. Pediatr. Psychol. 41, 983–992. 10.1093/jpepsy/jsw040 27189691

[B43] Van Dyk T. R. Zhang N. Combs A. Howarth T. Whitacre C. McAlister S.. (2019c). Feasibility and impact on daytime sleepiness of an experimental protocol inducing variable sleep duration in adolescents. PLoS ONE 14:e0218894. 10.1371/journal.pone.0218894 31226161 PMC6588251

[B44] Weber L. Kamp-Becker I. Christiansen H. Mingebach T. (2019). Treatment of child externalizing behavior problems: a comprehensive review and meta–meta-analysis on effects of parent-based interventions on parental characteristics. Eur. Child Adolesc. Psychiatry 28, 1025–1036. 10.1007/s00787-018-1175-3 29948228

[B45] Yoo S. S. Gujar N. Hu P. Jolesz F. A. Walker M. P. (2007). The human emotional brain without sleep—a prefrontal amygdala disconnect. Curr. Biol. 17, R877–R878. 10.1016/j.cub.2007.08.007 17956744

